# Maintenance of electrostatic stabilization in altered tubulin lateral contacts may facilitate formation of helical filaments in foraminifera

**DOI:** 10.1038/srep31723

**Published:** 2016-08-19

**Authors:** David M. Bassen, Yubo Hou, Samuel S. Bowser, Nilesh K. Banavali

**Affiliations:** 1 Laboratory of Computational and Structural Biology, Division of Genetics, Wadsworth Center, New York State Department of Health, PO Box 509, Albany, NY 12201-0509, USA; 2 Laboratory of Cellular and Molecular Basis of Diseases, Division of Translational Medicine, Wadsworth Center, New York State Department of Health, PO Box 509, Albany, NY 12201-0509, USA; 3 Department of Environmental Health Sciences, School of Public Health, State University of New York at Albany, USA; 4 Department of Biomedical Sciences, School of Public Health, State University of New York at Albany, USA

## Abstract

Microtubules in foraminiferan protists (forams) can convert into helical filament structures, in which longitudinal intraprotofilament interactions between tubulin heterodimers are thought to be lost, while lateral contacts across protofilaments are still maintained. The coarse geometric features of helical filaments are known through low-resolution negative stain electron microscopy (EM). In this study, geometric restraints derived from these experimental data were used to generate an average atomic-scale helical filament model, which anticipated a modest reorientation in the lateral tubulin heterodimer interface. Restrained molecular dynamics (MD) simulations of the nearest neighbor interactions combined with a Genalized Born implicit solvent model were used to assess the lateral, longitudinal, and seam contacts in 13-3 microtubules and the reoriented lateral contacts in the helical filament model. This electrostatic analysis suggests that the change in the lateral interface in the helical filament does not greatly diminish the lateral electrostatic interaction. After longitudinal dissociation, the 13-3 seam interaction is much weaker than the reoriented lateral interface in the helical filament model, providing a plausible atomic-detail explanation for seam-to-lateral contact transition that enables the transition to a helical filament structure.

Microtubules are cytoskeletal structures involved in multiple functions including cell division, transport of large cellular components such as chromosomes or vesicles, and whole cell motility^1^. Their dysfunction results in cardiac^2^ and neurodegenerative diseases^3^, and multiple anti-cancer agents exert their activity by binding to microtubules^4^. Their general architecture is shown by structural studies^5^,^6^ to be a cylindrical lattice of parallel protofilaments, which are themselves longitudinal chains of *αβ*-tubulin heterodimers, with neighboring protofilaments connected through lateral *α*-*α* and *β*-*β* tubulin contacts. The most commonly observed architecture is composed of 13 protofilaments with a longitudinal offset of 3 monomers between the first and thirteenth monomers at a “seam” interface, and is referred to as a “13-3” microtubule^7^. Alternative microtubule architectures are observed both *in vitro* and *in vivo*, and include protofilament numbers in the range of 8 to 20^8^. Alternate 11-3, 12-3, 14-3, 15-4, and 16-4 architectures have been characterized at a medium resolution to show the range of tolerance of lateral interactions to deformation, and the lack of lateral *α*-*β* contacts at the seam in the 15-4 and 16-4 architectures^9^.

The structural, mechanical, and dynamic properties of microtubules have been studied extensively using computational models^10^. Atomic models of microtubules were used along with implicit solvent calculations to estimate lateral and longitudinal interactions between tubulin dimers and protofilaments^11^. Explicit solvent MD simulations combined with Molecular Mechanics/Generalized Born Surface Area (MM/GBSA) calculation of inter-dimer lateral and longitudinal interaction energies for a periodic, infinitely long bovine 13-3 microtubule were used to understand residue-level contributions to overall microtubule stability^12^. A detailed free energy profile of tubulin heterodimer straight-bent conformational changes was determined, and its modulation by colchicine was studied, using explicit solvent MD simulations^13^. The energy landscape of unfolding of tubulin monomers and dimers, calculated using a self-organized polymer model, demonstrated the factors contributing to the intrinsic rigidity of tubulin^14^. A combination of atomistic simulations and coarse-grained analysis was used to understand how the nucleotide bound to the tubulin can affect structure and dynamics of a tubulin heterodimer^15^, a protofilament^16^, and the lateral and longitudinal interactions in microtubules^17^. Multiscale models using all-atom simulations and continuum mechanics could reproduce not only the Young’s modulus and persistence length of microtubules, but also the change in stiffness due to binding of ligands such as taxol^18^. Explicit solvent simulations on a full microtubule explained microtubule properties such as softening under extension, deformation under radial compression, and an asymmetric response to twisting in opposite directions^19^. The utility of such atomistic modeling for drug design was demonstrated by combining it with computational docking to identify a common binding site for antimitotic peptides on *β*-tubulin^20^, and also how dinitroaniline herbicides^21^ and taxol^22^ interact with microtubules. Multiscale computational modeling also showed that a compressed microtubule behaves as a rigid element system interconnected by a lateral and longitudinal elastic bond network, with the dissociation free energies for the lateral and longitudinal interactions being 6.9 and 14.9 kcal/mol, respectively^23^.

Foraminifera (forams) are aquatic protists that sample their environment through long, branched, and anastomosed pseudopodia, whose motility is dependent on microtubule networks^24^,^25^. Foram tubulin assemblies have been observed in the conventional 13-3 microtubule lattice and an unusual helical filament state that is thought to represent a storage or transport form of microtubule proteins^26^. This helical filament state can be reversibly induced by exposure to higher ionic concentrations of Ca or Mg, and is expected to be composed of strands of laterally connected *αβ*-tubulin heterodimers^27^. For such a helical filament to form, it has been postulated that certain foram tubulin sequence features allow lateral tubulin heterodimer interactions to be maintained while longitudinal tubulin heterodimer interactions are diminished^28^. The broad dimensions of these foram helical filaments have been measured^27^, but the structural rearrangements required at the atomic level or the underlying interactions that stabilize the helical filament state are not well-understood.

In the present study, an average atomic-scale structural model of a foram tubulin helical filament is generated based on a 13-3 bovine microtubule structure and experimentally measured foram helical filament coarse geometric parameters^27^. Restrained protein backbone simulations are performed on tubulin tetramer geometries to probe the electrostatic properties of specific neighboring heterodimer contacts in 13-3 microtubules and the helical filament model. The electrostatic interactions of the lateral contacts in the helical filament model are compared to those of lateral contacts in the 13-3 microtubule using Generalized Born (GB) implicit solvent model calculations. The electrostatic interactions of longitudinal contacts in the GTP-bound state, and lateral contacts at the 13-3 seam, are also estimated to better understand the electrostatic factors contributing to helical filament formation.

## Results

### Helical filament modeling

#### Coarse-grained structural models

**Figure 1:**
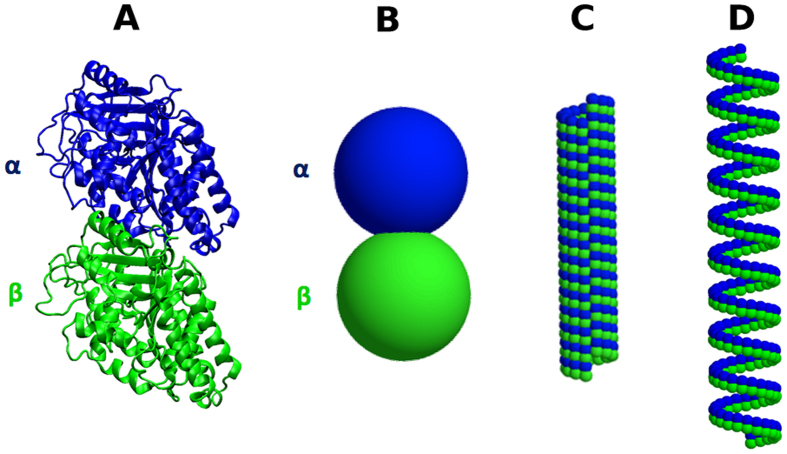
Modeling the helical filament state with a single particle representation of each tubulin monomer. (**A**) Detailed *αβ*-tubulin heterodimer structure, (**B**) Coarse grained model where every monomer is represented by a single point mass, (**C**) 13-3 architecture in this coarse-grained model, (**D**) Average helical filament architecture in this coarse-grained model.

The negative stain EM data provide geometric parameters of cylindrical diameter and distance between coils for foram helical filaments^27^ that are only at the resolution of individual tubulin *αβ*-heterodimers. A coarse-grained microtubule structural model consisting of two point masses representing each tubulin heterodimer allowed enforcement of the highly coarse-grained geometrical parameters using analogous cylindrical and longitudinal geometric restraints (illustrated in Fig. 1A,B). At this level, only the overall geometric features of 13-3 microtubules (Fig. 1C) and helical filaments (Fig. 1D) in the EM data can be reproduced. To reduce internal or lateral contact distortions due to the very coarse helical filament restraints, additional lateral distance, angle, and torsion restraints were incorporated based on the 13-3 architecture (listed in Supplementary Information Table 1). The minima in all these coarse-grained restraints are derived from the negative stain EM data for the helical filament or a known higher-resolution 13-13 microtubule structure^9^. Since the goal is simply generating a structure consistent with the experimental data, the force constants employed have no physical meaning. They can be as high as necessary, and were empirically assigned to a value that was sufficient to impose geometries close to the required parameter value.

The 13-3 architecture coarse-grained structural models were then optimized using a minimization and restrained MD simulations protocol to guide a smooth transition to a helical filament state. One basic assumption in this approach is that the helical filament state is composed of dimer strands instead of curved protofilaments, which is supported by low resolution EM data^27^. The initial 13-3 microtubules have a seam with *α*-*β* contacts, that are changed to *α*-*α* and *β*-*β* contacts in the helical filaments. This rearrangement of *α*−*β* seam interactions into *α*−*α* and *β*−*β* lateral interactions was enforced simply through the absence of *α*−*β* lateral restraints, and presence of direct *α*−*α* and *β*−*β* lateral restraints at the seam.

Explicit coarse-grained structural models for helical filaments consisting of 195 *αβ*-heterodimers spanning the entire experimentally expected range in cylindrical diameter and strand separation were generated at 0.1 nm increments. As shown in Supplementary Information Figure 1, the overall helical filament height variation can be predicted for these structural models. The observed values show that helical filaments gradually increase in height from the top left (large cylindrical radius and small longitudinal distance) to the bottom right (small cylindrical radius and large longitudinal distance). As is to be expected, the two parameters compensate for each other, i.e. the overall height can remain the same if both the cylindrical diameter and the longitudinal separation increase simultaneously. The overall height range (180–240 nm) indicates that helical filaments cannot be shorter than 13-3 microtubules, whose end-to-end distance should be around 135 nm for the 195 heterodimer containing models. This is true at least in the range of cylindrical diameter and longitudinal separation between dimer strands determined by negative stain EM methods^27^. The average helical filament geometry anticipated from the experimental data was chosen to postulate an atomic-detail helical filament structural model, as described below.

#### Atomic-detail structural model

To obtain an atomic-scale structural model of a foram helical filament from the highly coarse-grained structural model, three additional assumptions are necessary: (a) the axis of individual *α*-*β* heterodimers was assumed to remain aligned with the overall 13-3 microtubule helical axis; (b) the rotational orientation of the *α*-*β* heterodimers with respect to the central cylindrical axis was also assumed to remain consistent with that in the 13-3 microtubule; (c) the helical filaments are assumed to have a uniform cylindrical diameter. The first two assumptions are adopted to minimize the deviation from the known experimental 13-3 microtubule structure. The third assumption allows construction of a uniform idealized helical filament structure consistent with the average of the parameter range of the experimental negative stain EM data. These three assumptions together allow minimal deviation from the 13-3 structure, while enabling full compliance with the negative stain EM measurements. An expectation implicit in these assumptions is that the residues facing the lumen in the 13-3 microtubule will also face the lumen in the helical filament. Symmetry-breaking complicating factors such as a variable diameter within the same helical filament, or slightly differing helical axis orientations of individual tubulin heterodimers, are always possible. These can, however, be readily examined as perturbations of the initial idealized helical filament structure. Given the large geometric range of the negative stain EM helical filament measurements, it is also possible that there is no single “correct” helical filament structure analogous to the 13-3 microtubule structure, and that the reality can be better represented as a broad distribution of varying helical filament structures.

**Figure 2:**
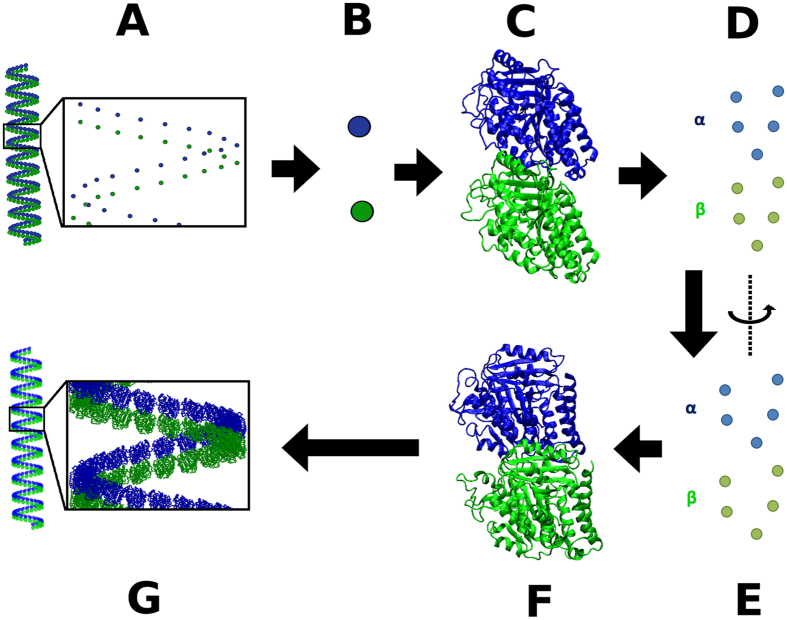
A workflow to predict an atomic-detail foram helical filament model. (**A**) Average coarse-grained helical filament model predicted using negative stain EM data; (**B**) Each tubulin heterodimer represented by two center-of-mass points; (**C**) Overlay of axially oriented foram tubulin monomer homology models on their corresponding center-of-mass; (**D**) Extraction of 10 reference C-*α* atoms from overlaid foram tubulin heterodimer homology model; (**E**) Cylindrical restraints reorient 10 reference C-*α* atoms with respect to central helical filament axis through rotation around individual heterodimer axis; (**F**) Overlay foram tubulin heterodimer homology model on 10 reference reoriented C-*α* atoms; (**G**) Atomic-detail helical filament model built using steps (**B**–**F**) for each coarse-grained foram tubulin heterodimer.

**Figure 3:**
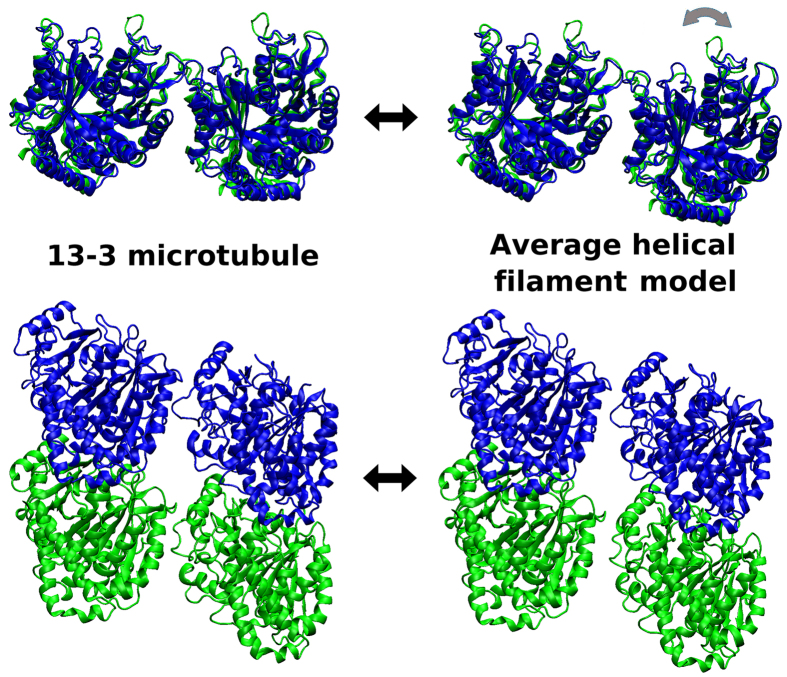
A comparison of top and side views indicating subtle differences between lateral interactions in a 13-3 microtubule and the average helical filament model.

An atomic-scale structural model was generated through the following steps (illustrated in Fig. 2): (a) a homology model for the foram *α*-*β* heterodimer was generated based on the bovine *α*-*β* heterodimer structure^6^; (b) each atomic-scale foram tubulin monomer homology model was translated to a coarse-grained monomer center-of-mass in the helical filament model; (c) cylindrical restraints were imposed on select conserved reference residue C-*α* atoms to enforce the same rotational orientation for them as the 13-3 architecture, and an optimization was performed. The restraint distance from the helical filament cylindrical axis was set to be *R*_*i*_ + *R*_*cyldiff*_, where *R*_*i*_ is the distance of the C-*α* atom from the 13-3 microtubule cylindrical axis, and *R*_*cyldiff*_ is the difference in cylindrical radius between the 13-3 microtubule and the average helical filament model; (d) the atomic-scale foram monomer homology model was overlaid onto these reoriented reference C-*α* atoms to obtain an atomic-scale helical filament model. It should be noted that this model is merely an atomic-scale projection of the average negative stain EM coarse grained helical filament geometric parameters with reasonable additional assumptions. Nevertheless, it provides a specific prediction for changes in lateral contacts in the transition from 13-3 microtubules to helical filaments that can be probed using atomic-scale MD methods. The details of the differences in the 13-3 and helical filament model inter-dimer lateral interfaces are shown in Supplementary Information Figure 2. The reorientation that occurs between Fig. 2C,F is illustrated in Supplementary Information Figure 3. The differences in the 13-3 and helical filament model inter-dimer lateral interfaces are also illustrated using targeted molecular dynamics (TMD) simulations between the two states in Supplementary Information videos 1 and 2, the RMSD differences during these TMD simulations are shown in Supplementary Information Figure 4. The static differences between neighboring lateral foram tubulin dimers in the 13-3 and average helical filament structures are shown in Fig. 3.

### Inter-dimer electrostatic interactions

**Figure 4:**
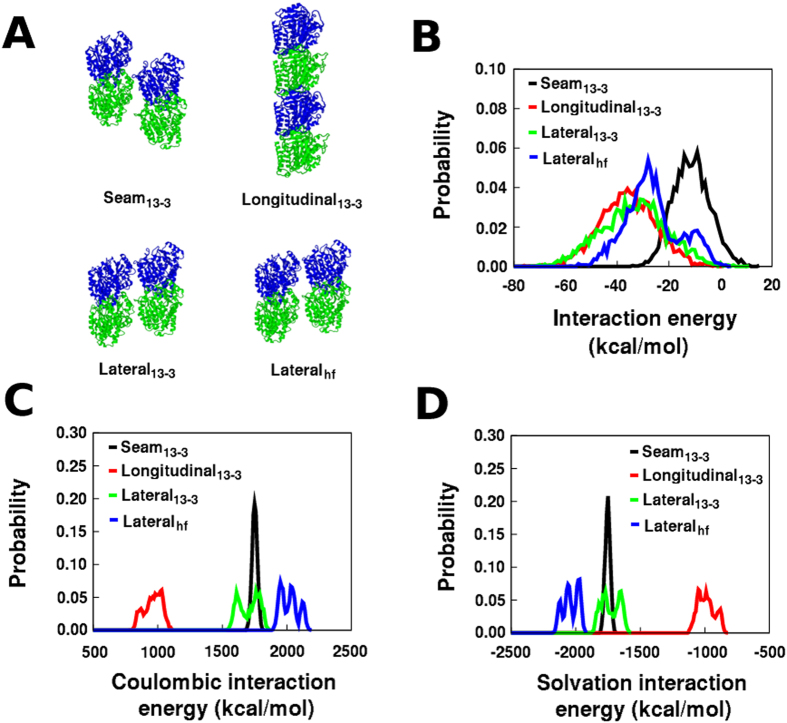
Foram tubulin inter-dimer interfaces and their electrostatic interaction energy distributions. (**A**) Four foram tubulin inter-dimer nearest-neighbor interfaces evaluated, (**B**) Overall electrostatic interaction energy distributions for the four interfaces, (**C**) Coulombic inter-dimer interaction energy distributions for the four interfaces, (**D**) Solvation electrostatic inter-dimer interaction energy distributions for the four interfaces. Interaction energies from all 5 simulations for each tetramer were pooled together, and the probability distributions were generated as histograms with a bin width of either 1 kcal/mol or 10 kcal/mol.

To estimate electrostatic interactions between relevant nearest neighbor contacts, three different foram tetramer models were excised from the 13-3 microtubule model (lateral contact, seam contact, longitudinal contact), and one foram tetramer lateral contact model was excised from the atomic-detail average helical filament model (shown in Fig. 4A). Five separate restrained MD simulations of each of these four models (20 total simulations of duration 1 ns each) were performed, and snapshots were extracted every 1 ps after 10 ps of equilibration. In these simulations, harmonic restraints of 1 kcal/mol/Å^2^ were applied on all tubulin protein backbone atoms to prevent larger structural variations, and to still capture the influence of protein sidechain dynamics. The dimer-dimer electrostatic interactions were then analyzed in the extracted snapshots using a Generalized Born simple switching (GBSW) implicit solvent model^29^ with an infinite pair-wise atomic distance cutoff for the electrostatic interactions. The solvent-screened inter-dimer electrostatic interaction energies obtained for each snapshot were then averaged in each independent simulation, and also pooled together and averaged, and are shown in Table 1. The opposing contributions of Coulombic electrostatic interaction and solvation electrostatic interaction energies, which are summed to get the inter-dimer interaction energies are shown in Table 2. The actual distributions for the overall electrostatic interaction energy and the opposing Coulombic and electrostatic solvation interaction energy components are shown in Fig. 4B–D.

The electrostatic interaction energies show some variation between the different simulations, but yield consistent averages and distributions when pooled. The final structure sidechain RMSD differences between simulations 1 and 3 for the 13-3 architecture, and simulations 1 and 4 for the helical filament average structure show that there are multiple variations that can lead to the inter-simulation differences in average interaction energies (Supplementary Information Figure 6). In 13-3 foram microtubules, the intra-protofilament longitudinal interaction between *α*-*β* heterodimers in the presence of GTP coordinated to two Mg ions is comparable to the inter-protofilament lateral interaction (only stronger by about 2 kcal/mol in the pooled average). A change in the lateral contacts from the 13-3 architecture to the average idealized helical filament model reduces the strength of the lateral interaction by about 7 kcal/mol, but this lateral contact still remains highly favorable (−27 kcal/mol). The source of the loss of longitudinal interactions in helical filaments is unclear, but once these interactions are lost, the adjustment of the seam to a lateral helical filament contact is favored by about 15 kcal/mol. This is consistent with the identification of the seam contact to be the weakest inter-dimer contact in 13-3 bovine microtubules^12^. A decoy foram tubulin tetramer structure obtained from the helical filament model prior to cylindrical restraint optimization was used to assess the interaction energy for an incorrect lateral inter-dimer interface as a negative control, and the average lateral interaction energy for this decoy structure (+15 kcal/mol) was estimated to be unfavorable (Supplementary Information Figure 7).

The lateral electrostatic interactions in the GTP-bound 13-3 bovine microtubule were estimated to be repulsive (73 kcal/mol)^12^, while these interactions are attractive in our foram 13-3 microtubules (−34.2 kcal/mol). Although the force fields, implicit solvent models, and other methodological details in these two estimations are distinct, this difference could be primarily due to the sequence distinctions between bovine and foram tubulin enabling greater electrostatic stabilization in foram tubulin lateral inter-dimer interactions. An examination of the trends in the Coulombic and solvation components (Fig. 4C,D) shows that low dielectric environment interactions between foram tubulin heterodimers in all interfaces are intrinsically repulsive, and the solvent screening in a high-dielectric environment reduces this repulsion to help in the stabilization of the foram tubulin inter-dimer contacts. Environmental electrostatic perturbations could therefore trigger foram helical filament formation both through direct local interactions, as well as a distributed change in these large favorable solvation contributions.

## Discussion

In the negative stain EM analysis, the overall length of helical filaments was surmised to be smaller than the 13-3 microtubules that they originated from^27^. This was based on the observation that the overall length of helical filament fragments was shorter than the length of the corresponding gap in the originating microtubule in the negative stain specimens^27^. This is consistent with a larger cylindrical diameter, but not with a greater longitudinal inter-dimer distance in the helical filaments. The coarse-grained structural models clarify this issue directly, and show that the measured range of cylindrical diameter and longitudinal distance parameters can only allow for helical filaments longer than 13-3 microtubules. Since these models rely on only the negative stain EM data, and account for the size of individual *α*-*β* heterodimers, it seems that there is either relative motion between the separated 13-3 microtubules and the helical filaments in the negative stain preparations, or a loss of some tubulin dimers during the formation of the separated helical filaments. Further experimental evidence, ideally a 3D reconstruction of helical filaments using cryo-EM techniques, should be able to confirm this prediction.

**Figure 5:**
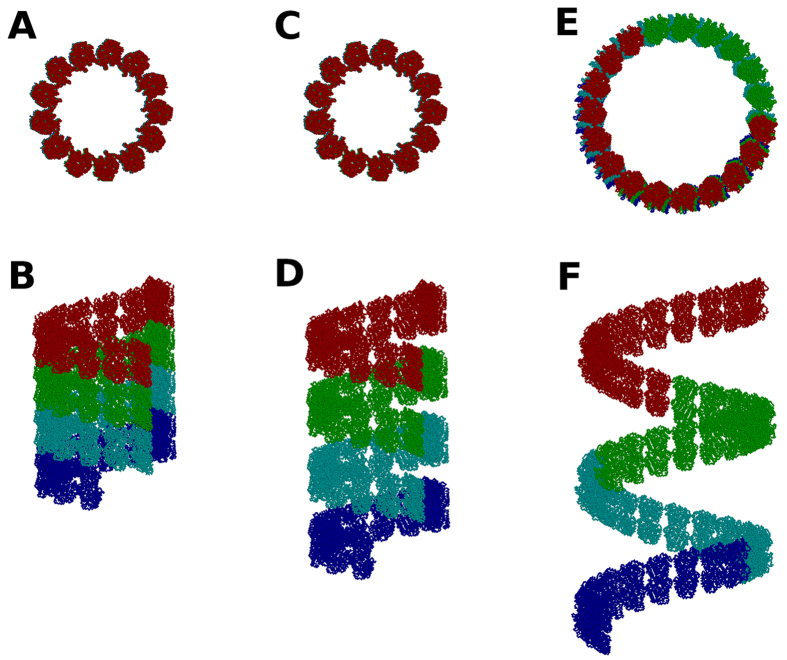
Tubulin assembly structures consisting of 50 tubulin heterodimers to illustrate structural changes required to form helical filaments consistent with the negative stain EM data. (**A**,**B**) 13-3 microtubule assembly, (**C**,**D**) helical filament with 13-3 lateral interface maintained, but not compliant with the geometric parameter range of the negative stain EM data, (**E**,**F**) helical filament representing the average of the geometric parameter range of the negative stain EM data, but with lateral interface changed as compared to 13-3 architecture. Tubulin dimers 1–13 are shown in red, tubulin dimers 14–26 are shown in green, tubulin dimers 27–39 are shown in cyan, and the rest of the tubulin dimers are shown in blue.

The main requirement for the formation of laterally connected tubulin helical filaments is the complete dissociation of the longitudinal *β*-*α* GTP-mediated contacts. A change in lateral contacts is not required, except at the seam, where *α*-*β* and *β*-*α* lateral contacts need to transition to regular lateral *α*-*α* and *β*-*β* contacts, respectively. Once these two changes have occurred, a seamless helical filament can be formed with the same lateral contacts and cylindrical diameter as 13-3 microtubules, but with a separation between longitudinally closest tubulin dimers equal to the height of one tubulin monomer (about 5 nm). This is illustrated using three 50-dimer tubulin assembly structures in Fig. 5. The 13-3 microtubule structure is shown in Fig. 5A (top view) and Fig. 5B (side view), a helical filament structure that maintains the lateral contacts the same as the 13-3 structure is shown in Fig. 5C (top view) and Fig. 5D (side view), and the helical filament structure consistent with the average of the negative stain EM measurements is shown in Fig. 5E (top view) and Fig. 5F (side view). Successive neighboring groups of 13 dimers (one turn in the 13-3 structure) are colored differently to illustrate the overall distinctions between the three structural assemblies. It can be seen that the cylindrical diameter remains unchanged between the 13-3 microtubule (Fig. 5A) and the helical filament structure that maintains its lateral contacts (Fig. 5C). The negative stain EM data on actual foram helical filaments^27^ do not agree with the structure shown in Fig. 5C,D. These data indicate both an increase in cylindrical diameter and longitudinal separation, which only a change in lateral contacts from those seen in 13-3 microtubules can accommodate. Such flexibility in lateral tubulin contacts is not inherently surprising. Similar lateral rearrangement occurs in taxol or taxotere-DMSO stabilized alternate 11-3, 12-3, 14-3, 15-4, and 16-4 bovine microtubule architectures^9^. In sea urchin sperm flagella axonemal microtubule doublets, an ellipsoid tubule is formed that also requires some lateral adjustment^30^. In high zinc concentrations, even greater lateral adjustment is required to form tubulin sheets instead of cylindrical microtubules^31^.

The predicted average helical filament atomic-detail model offers just one possibility among many for a change in lateral contacts consistent with the broad range of the negative stain EM data. The foram tubulin sequences may encode for general lateral contact flexibility or a more favored specific alternate lateral contact or both. Structural variations in the internal *α*-*β* heterodimer structure do not seem to be required for such flexibility, but modest internal adjustments could provide additional stabilization for such helical filament structures. Partition homogeneity analysis suggests that foram *α*-tubulin sequences do not differ much from other *α*-tubulin sequences, but foram *β*-tubulin sequences do show higher divergence, especially in lateral contact and taxol-binding regions^28^. Phylogenetic analysis of 119 new *β*-tubulin sequences also suggests positive selection of three specific *β*-tubulin residues: Val184 at the longitudinal interface, and Asp297 and Ser302 at the lateral interface, in tubulin inter-dimer contacts in forams^32^. Although the approach used here relies on very coarse-grained experimental data, it does provide a route to generate specific structural hypotheses to guide structure determination studies or interpret phylogenetic sequence data. The present approach could also be used to anticipate the general features of other microtubule deformations for which high-resolution data collection is difficult or costly.

## Methods

### Atomic-scale 13-3 foram microtubule

The 3.5 Å resolution electron crystallography model of a bovine tubulin heterodimer (PDB ID: 1JFF^6^), was used as a template to generate homology models for tubulin heterodimers. A region of missing bovine *α*-tubulin loop residues was homology modeled using alignment with a *β*-tubulin sequence:

QMPSDKTIGGGDDSFNTFFSETGAGKH- (*α*-tubulin sequence)

SYHGDSDL-QLERINVYYNEAAGNKYV (*β*-tubulin sequence)

The assumption was that the lowest energy structure is similar for this region in both monomers, with the region in *α*-tubulin not resolved experimentally due to its greater flexibility. The *α*- and *β*-tubulin sequences from the foram species *Reticulomyxa filosa* were aligned to the bovine sequence using ClustalX^33^. Homology models with all alignments were generated using the program MODELLER^34^ with the DOPE and GA341 energy functions.

The foram tubulin homology models were assessed by a few different Model Quality Assessment Programs (MQAPs) (summarized in Supplementary Information Table 2). A Qmean score was obtained from the Swiss-Model Qmean server^35^ (http://swissmodel.expasy.org/qmean/cgi/index.cgi). This Qmean score is a global score with values ranging between 0 and 1, with 1 representing greatest accuracy. The Swiss-Model Z-score relates this Qmean score to the scores of a non-redundant set of high-resolution X-ray structures of similar size, and its ideal values are close to 0. ProSA-Web Z-scores^36^ were obtained using the ProSA-Web server (https://prosa.services.came.sbg.ac.at/prosa.php). These Z-scores are mostly within the range −7 to −12 for protein crystal structures. The Protein Structure Validation Server (PSVS1.5)^37^ was used to obtain raw scores for Verify3D^38^ and Procheck G-factor^39^ assessments. As with the other Z-scores, values closer to 0 indicate models that better agree with a high quality protein crystal structure dataset. A Molprobity score^40^ was obtained using the Joint Center for Structural Genomics’ Quality Control Check version 3.1 (http://smb.slac.stanford.edu/jcsg/QC/). A meta-assessment was carried out using MetaMQAP^41^ (https://genesilico.pl/toolkit/unimod?method=MetaMQAPII) to obtain an MetaMQMAPII score. Both the Molprobity and MetaMQMAPII scores reflect the expected value of the X-ray resolution in Å given the scores for the model. All these assessments are consistent with the homology models being roughly as accurate as the bovine tubulin structure (PDB ID: 1JFF^6^, 3.5 Å resolution) used as a template. This is to be expected given the relatively high sequence identity between the bovine and foram tubulin sequences, which is 82% for *α*-tubulin and 45% for *β*-tubulin (alignment shown in Supplementary Information Figure 5).

The GDP molecule at the longitudinal interface between tubulin heterodimers was replaced by a GTP molecule with 2 neighboring Mg ions to enable plausible electrostatic calculation of especially the stable GTP-mediated 13-3 microtubule longitudinal interaction. A high resolution structure of GTP with two neighboring Mg ions was overlaid (PDB ID: 1L2X, 1.25 Å resolution^42^) was overlaid on the GDP molecule using a least-squared superposition^43^ of the guanine base non-hydrogen atoms. With the protein non-hydrogen atoms held fixed, the added GTP and Mg ions were restrained by a harmonics restraint with a force constant of 10 kcal/mol, and minimized using a Steepest Descent (SD) algorithm for 5000 steps with an energy change tolerance of 0.001 kcal/mol. The harmonic restraint force constant was reduced to 1 kcal/mol and another 5000 steps of SD minimization with an energy change tolerance of 0.001 kcal/mol was performed. Finally, the whole system, including the protein, was minimized under harmonic restraints with force constant 1 kcal/mol on all non-hydrogen atoms for 5000 SD steps with an energy change tolerance of 0.001 kcal/mol to obtain a minimized starting structure to be used for further calculations. This limited GTP modeling is not expected to generate an accurate internal conformation of the GTP-state of the foram tubulin dimer. It is merely anticipated to minimally incorporate the large expected electrostatic effects of the presence of the third phosphate and divalent ions in this nucleotide binding pocket. Each minimized foram tubulin heterodimer model was then overlaid onto the bovine tubulin heterodimers in the 13-3 microtubule structure derived from an 8 Å cryo-EM map^9^ using C-*α* atoms of identically conserved residues to obtain an atomic-detail foram 13-3 microtubule model.

### Coarse grained foram helical filament

Microtubule coarse grained structural models with each tubulin monomer represented as a single point mass particle were generated using the Molecular Mechanics (MM) program CHARMM (version c35b3)^44^,^45^. For this, the cartesian coordinates for the center-of-mass of each monomer in the atomic scale foram 13-3 microtubule model were assigned as initial coordinates of a point mass representing that monomer. The interactions between monomers were maintained using distance, angular, and torsional harmonic restraints applied using the Miscellaneous Mean Field Potential (MMFP) module in CHARMM. These restraints used the 13-3 microtubule structure to obtain the minimum energy values, and ad hoc force constants required to maintain these geometric features. These parameters are shown in Supplementary Information Table 1.

To generate a helical filament from the initial 13-3 microtubule structure, additional restraints derived from the published parameters for the geometrical properties of the foram helical filaments^27^ were applied. The *β*_*n*_ : *α*_*n*+14_ lateral seam interaction observed in 13-3 microtubules is incompatible with helical filament formation, but the *α*_*n*_ : *β*_*n*+1_ lateral seam interaction could possibly be maintained in the helical filament. Maintaining just this single interaction between lateral neighbors is, however, less likely than undergoing a relatively small longitudinal shift to form a more stable double interaction between *α*_*n*_ : *α*_*n*+1_ and *β*_*n*_ : *β*_*n*+1_ monomers. Harmonic restraints for the lateral seam interactions (*α*_*n*_ : *β*_*n*+1_ and *β*_*n*_ : *α*_*n*+14_) were therefore replaced by regular lateral interactions (*α*_*n*_ : *α*_*n*+1_ and *β*_*n*_ : *β*_*n*+1_).

The cylindrical diameter is increased and longitudinal interactions between heterodimers are expected to be lost in helical filament formation^27^. The longitudinal *β*_*n*_ : *α*_*n*+13_ restraints were therefore removed, and a cylindrical MMFP restraint enforcing a monomer distance from the microtubule axis was used to increase the diameter of the microtubule to the required extent. As can be expected, this increase in diameter caused an average protofilament registry increase from 13 to 21 due to sliding of the microtubule heterodimer strands relative to one another. This still cylindrical structure was then converted to a helical filament structure by using gradually increasing longitudinal restraints between *α*_*n*_ : *β*_*n*_ and *α*_*n*+21_ : *β*_*n*+21_ tubulin heterodimers (from 107 Å to 193 Å) and *α*_*n*_ : *β*_*n*_ and *α*_*n*+43_ : *β*_*n*+43_ tubulin heterodimers (from 214 Å to 386 Å). The second order longitudinal restraints between *α*_*n*_ : *β*_*n*_ and *α*_*n*+43_ : *β*_*n* +43_ tubulin heterodimers were used to maintain uniformity in the lengthening throughout the helical filament.

All coarse-grained structural models were generated using the following optimization protocol in the presence of the restraints: (a) 2000 steps of Steepest Descent minimization to a tolerance of 0.001 kcal/mol; (b) 1000 steps of constant volume and temperature (NVT) dynamics at 100 Kelvin; (c) another 2000 steps of Steepest Descent minimization to a tolerance of 0.001 kcal/mol. The same procedure was used to produce a series of helical filament models for the entire range of longitudinal distance and cylindrical radii uncertainty reported experimentally^27^. As shown in Supplementary Information Figure 1, these models were also analyzed for their overall length, characterized as the end-point mass coordinates projected onto the microtubule longitudinal axis, with an adjustment for the internal longitudinal dimensions of the monomers themselves.

### Atomic scale foram helical filament

As a first step to reconstruct approximate atomic scale helical filament models, *α*- and *β*-monomers from a dimer structure extracted from the 13-3 foram microtubule structure were translated such that their center-of-mass overlaid with the monomer center-of-mass in the coarse grained helical filament structure. This yielded an atomic scale helical filament with an incorrect orientation of the monomers along their vertical axis, which was still parallel to the microtubule cylindrical axis (Supplementary information Figure 3B). To enforce an orientation of the monomers similar to the one in the 13-3 microtubule, 10 reference C-*α* atoms were extracted (Fig. 2D). These reference point C-*α* atoms belonged to conserved residues spread through the heterodimer (R121, K304, V324, P364, D424 in *α*-tubulin and P86, V126, G146, P311, F415 in *β*-tubulin). The specific residues chosen are arbitrary, and can be easily varied without consequence.

Rigid internal restraints were used to lock the relative position of the 10 C-*α* points in each dimer together, while corresponding lateral and longitudinal restraints between monomers are also maintained. To reorient these atoms correctly with respect to the microtubule cylindrical axis, cylindrical restraints were applied to position them at an appropriate distance from this axis. This distance was computed as a sum of the original distance of these points from the axis in the 13-3 architecture plus a constant offset due to the uniform increase in cylindrical diameter in the conversion to helical filaments. This set of 10 restraints, applied in concert for all 195 dimers, rotates the 10 reference points in each dimer to orientations consistent with their original orientations with respect to the central microtubule axis in the 13-3 structure. For this optimization, the following minimization and dynamics protocol was used: (a) 1000 steps of Steepest Descent minimization followed by 1000 steps of Adopted Basis Newton Raphson minimization, both to a tolerance of 0.001 kcal/mol; (b) 1000 steps of constant volume and temperature (NVT) dynamics at 100 Kelvin; (c) another 1000 steps of Steepest Descent minimization followed by 1000 steps of Adopted Basis Newton Raphson minimization, both to a tolerance of 0.001 kcal/mol. The initial optimization was performed with weak lateral restraint force constants of 1 kcal/mol/Å^2^. A second optimization was performed with stronger lateral restraint force constants of 900 kcal/mol/Å^2^. The restraints used in this reorientation are listed in Supplementary Information Table 3. Atomic-detail models were reconstructed by least-squared superposition^43^ of the foram monomer homology models onto the 10 reference C-*α* atoms. The foram sequences present in the final model are *α*-tubulin CAA65329 and *β*-tubulin CAA65332.

### Electrostatic interaction energy estimation

All tetramer MD simulations were performed using the program CHARMM (version c35b3)^44^,^45^, and the CHARMM22 protein force field^46^, and CHARMM27 force field parameters for GTP^47^,^48^. Four inter-dimer interfaces were studied (Fig. 4A): 13-3 seam, 13-3 longitudinal, 13-3 lateral, and helical filament lateral. All tetramer system models were generated using CHARMM-GUI^49^, the protein backbone non-hydrogen atoms were harmonically restrained with a force constant of 1 kcal/mol/*Å*^2^, and the system was minimized using 500 steps of Steepest Descent (SD) minimization with a convergence cutoff of 0.001 kcal/mol. Electrostatic and Lennard-Jones (LJ) interaction cutoffs of 16 Å were used with non-bond interaction lists maintained and heuristically updated out to 20 Å. Keeping protein backbone harmonic restraints in place, 5 simulations were performed for each tetramer system, each with a different random seed for distributing initial atomic velocities. A timestep of 1 femtosecond was used, and each simulation was run using the leapfrog integrator for 1 ns at a temperature of 300 K.

The inter-dimer interaction energies were assessed by the Generalized Born simple switching (GBSW)^29^ implicit solvent model using the protein^50^ and nucleic acid^51^ Born radii. A total of 990 snapshots from the final 990 ps of each simulation were examined, resulting in 4950 individual interaction energy estimations for each tetramer system. The GBSW electrostatic solvation energy was computed using a smoothing length of 0.6 Å, a nonpolar surface tension coefficient of 0.03 kcal/mol/*Å*^2^, a lookup table grid spacing of 1.5 Å, a solvent dielectric constant set to 80, and a protein dielectric constant set to 1. The Coulombic interactions were calculated using an infinite cutoff for non-bonded interactions. The interaction energy was computed for each structural snapshot as *E*_*tetramer*_−*E*_*dimer*1_−*E*_*dimer*2_, where *E*_*tetramer*_ is the energy of the tetramer, *E*_*dimer*1_ is the energy of the first dimer, and *E*_*dimer*2_ is the energy of the second dimer. This interaction component was calculated for both the Coulombic energy and the GBSW electrostatic solvation energy. The electrostatic interaction energy between two tubulin dimers was then computed as the sum of the Coulombic and electrostatic solvation interaction energies.

Molecular pictures were produced using VMD^52^ or Rasmol^53^, graphs were made using gnuplot version 4.4 (http://www.gnuplot.info) and compiled using GIMP version 1.2 (http://www.gimp.org) software; XSEDE resources^54^ were used for simulations.

## Additional Information

**How to cite this article**: Bassen, D. M. *et al*. Maintenance of electrostatic stabilization in altered tubulin lateral contacts may facilitate formation of helical filaments in foraminifera. *Sci. Rep.* **6**, 31723; doi: 10.1038/srep31723 (2016).

## Supplementary Material

Supplementary Information

Supplementary Vidoe S1

Supplementary Video S2

## Figures and Tables

**Table 1:** Average inter-dimer electrostatic interaction energies in foram 13-3 and helical filament nearest-neighbor tetramer models.

Simulation	1	2	3	4	5	Pooled
Seam_13−3_	−11.6 (5.9)	−4.8 (6.2)	−13.6 (6.9)	−13.4 (5.8)	−15.0 (5.8)	−11.8 (7.1)
Longitudinal_13−3_	−31.6 (10.4)	−39.7 (9.4)	−29.7 (10.0)	−42.7 (10.5)	−37.6 (9.0)	−36.5 (10.9)
Lateral_13−3_	−19.4 (8.7)	−49.8 (7.9)	−39.7 (7.3)	−30.8 (8.1)	−31.0 (7.2)	−34.2 (12.8)
Lateral_*hf*_	−38.0 (6.8)	−28.5 (5.0)	−29.9 (6.6)	−10.9 (4.4)	−27.2 (4.7)	−27.0 (10.5)

Standard deviations are shown in parentheses. Helical filaments are indicated by the subscript “hf” and 13-3 microtubules by the subscript “13-3”. All values are in kcal/mol.

**Table 2:** Average inter-dimer Coulombic and electrostatic solvation interaction energy contributions in foram 13-3 and helical filament tetramer models.

Simulation	1	2	3	4	5	Pooled
Seam_13–3_, *E*_*Coul*_	1751.8 (20.3)	1741.0 (15.5)	1745.8 (15.7)	1730.8 (19.2)	1761.6 (16.8)	1744.8 (20.2)
Seam_13–3_, *E*_*solv*_	−1763.4 (17.9)	−1745.8 (13.7)	−1759.4 (13.2)	−1744.1 (16.3)	−1776.6 (14.8)	−1756.5 (19.4)
Longitudinal_13–3_, *E*_*Coul*_	1010.4 (28.3)	856.5 (23.6)	962.4 (28.3)	930.0 (27.6)	1023.7 (33.6)	957.4 (67.2)
Longitudinal_13–3_, *E*_*solv*_	−1041.9 (29.6)	−896.2 (20.8)	−992.1 (26.7)	−972.7 (25.3)	−1061.3 (31.5)	−993.9 (64.5)
Lateral_13–3_, *E*_*Coul*_	1751.1 (34.2)	1605.1 (22.3)	1624.5 (33.7)	1732.3 (37.2)	1791.8 (29.9)	1700.1 (79.4)
Lateral_13–3_, *E*_*solv*_	−1770.5 (29.5)	−1654.8 (18.3)	−1664.2 (30.5)	−1763.0 (34.7)	−1822.8 (27.3)	−1734.3 (70.7)
Lateral_*hf*_, *E*_*Coul*_	1943.2 (21.1)	1953.9 (21.8)	2016.2 (26.2)	2118.5 (18.0)	2044.6 (17.7)	2015.5 (67.1)
Lateral_*hf*_, *E*_*solv*_	−1981.2 (17.9)	−1982.4 (20.0)	−2046.1 (23.9)	−2129.4 (16.2)	−2071.8 (16.0)	−2042.5 (59.0)

Standard deviations are shown in parentheses. *E*_*Coul*_: Coulombic interaction energies, *E*_*solv*_: electrostatic solvation interaction energies. All values are in kcal/mol.
