# Supplementary material for: Maintenance of electrostatic stabilization in altered tubulin lateral contacts may facilitate formation of helical filaments in foraminifera
Source: Sci Rep. 2016 Aug 19;6:31723. doi: 10.1038/srep31723 (PMC4990898; doi:10.1038/srep31723)
Supplement: Supplementary Information [file srep31723-s1.pdf]

Supplementary Information: Maintenance of electrostatic stabilization in altered tubulin lateral contacts may facilitate formation of helical filaments in foraminifera

David M. Bassen, Yubo Hou, Samuel S. Bowser*, Nilesh K. Banavali*

* corresponding authors: samuel.bowser@health.ny.gov, nilesh.banavali@health.ny.gov

Scientific Reports

Visualizing change in lateral interaction between the 13-3 and helical filament state

To visually demonstrate the subtle lateral inter-dimer reorientation required to change from a 13-3 microtubule structure to a helical filament structure, ten short targeted molecular dynamics simulations were performed pulling the lateral 13-3 tetramer structure to the lateral helical filament tetramer structure, and vice versa. The simulation methods were the same as for the interaction energy estimations, except that the protein backbone non-hydrogen atoms were not harmonically restrained. Instead, the protein backbone atoms of only one tubulin dimer were fixed. A very strong Root Mean Square Deviation (RMSD) restraint (1000000 kcal/mol/$\mathring{A}^2$ was imposed on all non-hydrogen atoms to change in 0.1 Å increments from one state to another, with 500 steps of Steepest Descent minimization, and a 1000 steps of dynamics at each step. As can be seen in the two Supplementary Information movies, this led to motion of the second tubulin dimer relative to the fixed first tubulin dimer from the 13-3 lateral orientation to the helical filament lateral orientation, and back.

Additional Files

Helical filament model

Pdb format file with helical filament Cα atom model corresponding to the average cylindrical diameter and longitudinal separation in the experimental data. Can be viewed using open source molecular visualization programs (e.g. VMD).

Movie 1

Mov format file (mt133_to_hf_top.mov) showing the top view for five targeted molecular dynamics (TMD) forward and backward simulations between lateral contacts in a 13-3 microtubule and lateral contacts in a helical filament.

Movie 2

Mov format file (mt133_to_hf_side.mov) showing the side view for five targeted molecular dynamics (TMD) forward and backward simulations between lateral contacts in a 13-3 microtubule and lateral contacts in a helical filament.

Table 1: **Coarse-grained model restraint parameters obtained from the 13-3 microtubule**

Distances	Minimum value (Å)	K_b (kcal/mol/Å^2)
Lateral $(\alpha_n : \alpha_{n+1})$	40.9	10.0
Lateral $(\beta_n : \beta_{n+1})$	52.2	10.0
Lateral seam $(\alpha_n : \beta_{n+1})$	52.7	0.0
Lateral seam $(\beta_n : \alpha_{n+14})$	51.7	0.0
Internal heterodimer $(\alpha_n : \beta_n)$	52.2	10.0
Longitudinal $(\beta_n : \alpha_{n+13})$	193.0	10.0
Longitudinal $(\beta_n : \alpha_{n+27})$	386.0	10.0
Angles	Minimum value (degrees)	K_a (kcal/mol/radian^2)
$(\alpha_n : \alpha_{n+1} : \beta_{n+1})$	100.96	10.0
$(\alpha_n : \beta_n : \beta_{n+1})$	100.93	10.0
Dihedrals	Minimum value (degrees)	K_d (kcal/mol/radian^2)
$(\alpha_n : \beta_n : \beta_{n+1} : \alpha_{n+1})$	0.31	20.0

A harmonic restraint of the form $K_x(x - x_{min})^2$ was used, where K_x is the force constant of the restraint, x_{min} is the minimum energy value of the restrained parameter, and x represents either the distance (b), angle (a), or dihedral (d) parameters.

Table 2: **Assessment of the foram tubulin heterodimer homology model**

Method	α-tubulin	β-tubulin
Swiss Model (Qmean score)	0.52	0.47
Swiss Model (Z-score)	-2.93	-3.53
ProSA-web (Z-score)	-8.98	-8.37
Verify3D	0.46	0.31
Procheck G-factor	-0.28	-0.26
Molprobity	3.33	3.48
MetaMQMAPII	3.44	3.76

Table 3: **Restraint parameters for the intermediate coarse-grained helical filament model**

Distances	Minimum value (Å)	K_b (kcal/mol/Å^2)
1-2	27.14	900.0
2-3	32.82	900.0
3-4	30.90	900.0
4-5	22.63	900.0
5-6	32.45	900.0
6-7	35.86	900.0
7-8	28.60	900.0
8-9	14.80	900.0
9-10	26.38	900.0
Angles	Minimum value (Å)	K_b (kcal/mol/Å^2)
1-2-3	87.7	900.0
2-3-4	89.9	900.0
3-4-5	79.8	900.0
4-5-6	87.1	900.0
5-6-7	94.8	900.0
6-7-8	53.0	900.0
7-8-9	55.6	900.0
8-9-10	149.8	900.0
Dihedrals	Minimum value (Å)	K_b (kcal/mol/Å^2)
1-2-3-4	65.4	450.0
2-3-4-5	2.9	450.0
3-4-5-6	98.8	450.0
4-5-6-7	299.8	450.0
5-6-7-8	136.1	450.0
6-7-8-9	54.0	450.0
7-8-9-10	15.1	450.0
Lateral	Minimum value (Å)	K_b (kcal/mol/Å^2)
3_n-5_{n+1}	22.2	1.0 or 900.0
7_n-6_{n+1}	20.7	1.0 or 900.0
3_n-6_{n+1}	46.9	1.0 or 900.0
7_n-5_{n+1}	35.6	1.0 or 900.0
Cylindrical	Radius (Å)	K_b (kcal/mol/Å^2)
1	148.9	1.0
2	136.5	1.0
3	158.6	1.0
4	180.7	1.0
5	164.6	1.0
6	167.1	1.0
7	160.7	1.0
8	179.3	1.0
9	165.0	1.0
10	140.1	1.0

A harmonic restraint of the form $K_x(x - x_{min})^2$ was used, where K_x is the force constant of the restraint, x_{min} is the minimum energy value of the restrained parameter, and x represents either distance (b), angle (a), or dihedral (d) parameters. The numbers specifying the restraints correspond to the 10 reference points in the intermediate coarse-grained model of each dimer. These points correspond to actual C-α atoms in the dimer as follows: 1 - V324 (α-tubulin), 2 - P364 (α-tubulin), 3 - R121 (α-tubulin), 4 - D424 (α-tubulin), 5 - K304 (α-tubulin), 6 - P311 (β-tubulin), 7 - V126 (β-tubulin), 8 - F415 (β-tubulin), 9 - G146 (β-tubulin), 10 - P86 (β-tubulin).

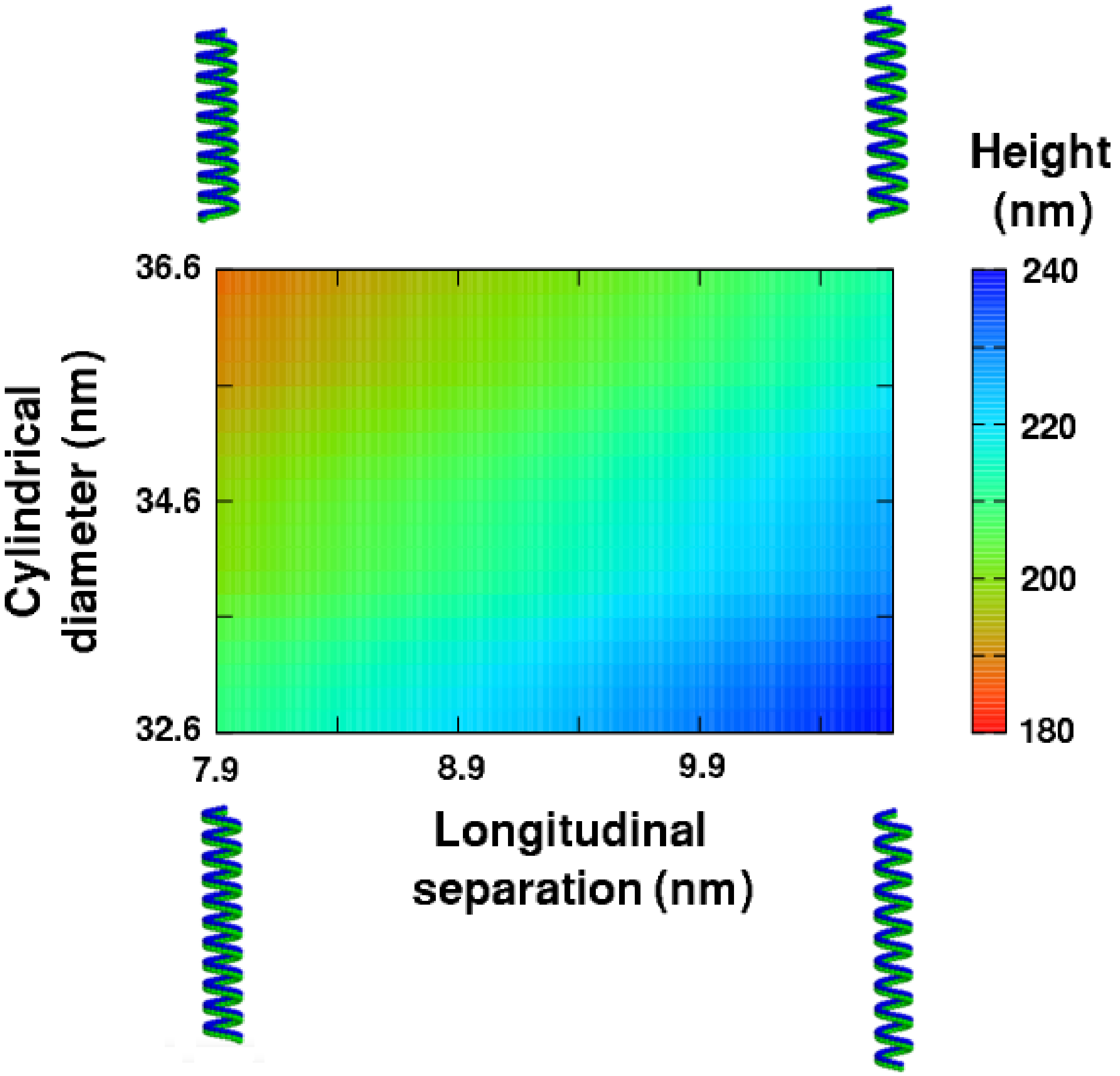

Figure 1: **Height variation in the experimentally derived 2D range of cylindrical radius and longitudinal distance between dimer strands in the foram helical filaments.** The four 195 dimer helical filament images depict the structures at the four extremes of this 2D space. All helical filament structures are longer than the original 195 dimer 13-3 microtubule, whose length is around 135 nm.

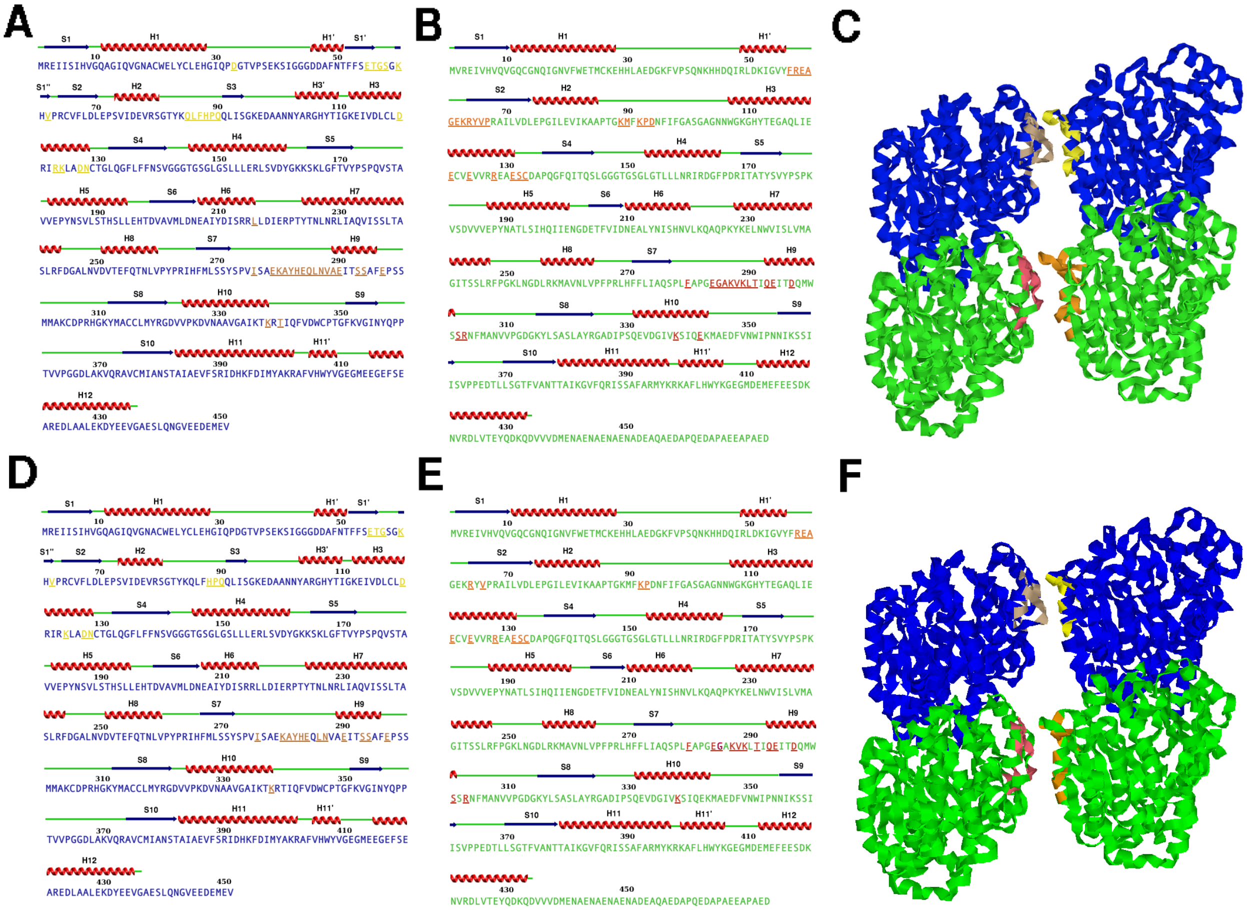

Figure 2: **Comparison of α-α and β-β interacting residues in 13-3 microtubule and the average helical filament model.** A. Sequence and secondary structure location of residues within 5 Å of neighboring dimer in the 13-3 microtubule for α-tubulin shown underlined and colored yellow and tan; B. Sequence and secondary structure location of residues within 5 Å of neighboring dimer in the 13-3 microtubule for β-tubulin shown underlined and colored orange and pink; C. Residues within 5 Å of neighboring dimer in a 13-3 microtubule tetramer structure; D. Sequence and secondary structure location of residues within 5 Å of neighboring dimer in the helical filament for α-tubulin shown underlined and colored yellow and tan; E. Sequence and secondary structure location of residues within 5 Å of neighboring protofilament in the helical filament for β-tubulin shown underlined and colored orange and pink; F. Residues within 5 Å of neighboring dimer in a helical filament tetramer structure. Secondary structure elements indicated in sequences show α-helices in red, β-sheets in blue, and everything else in green. The helical filament structures are without any additional minimization, i.e. the internal structure of the monomers is the same as for the 13-3 microtubule state.

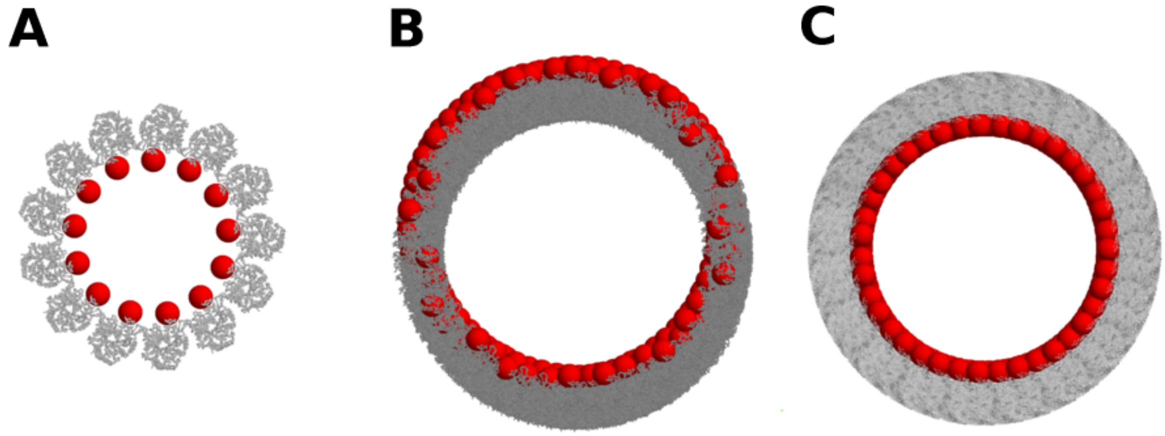

Figure 3: **Reorientation of tubulin monomers in the average helical filament model using cylindrical restraints.** A. top view of 13-3 microtubule showing known orientation of monomers, B. top view of initial helical filament model showing random orientation of monomers with respect to central cylindrical axis, C. top view of final helical filament model showing monomers maintaining their orientation with respect to central axis same as the 13-3 microtubule architecture. Orientation indicated using a reference red spacefill residue that faces the lumen of the 13-3 cylindrical microtubule (Lys43).

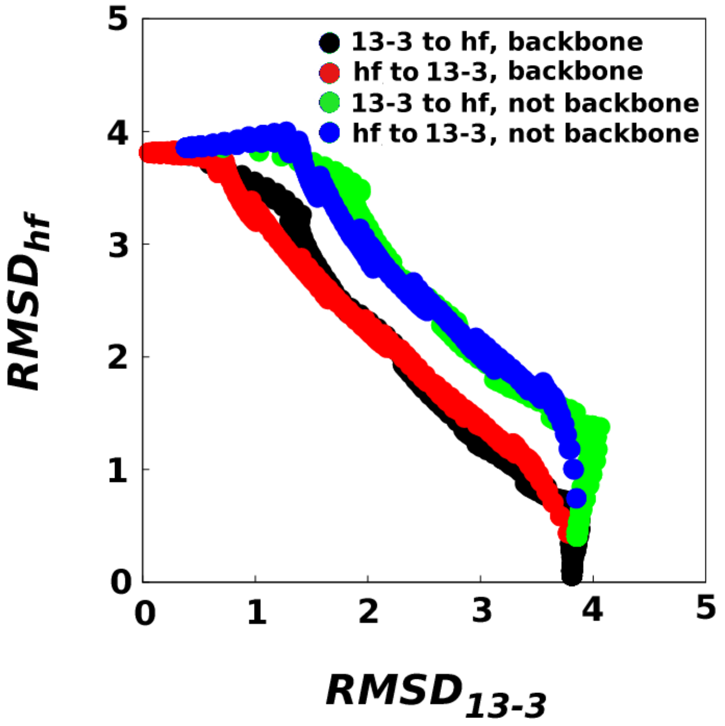

Figure 4: **Root Mean Square Deviation (RMSD) for TMD simulations between 13-3 lateral contacts and helical filament lateral contacts.**

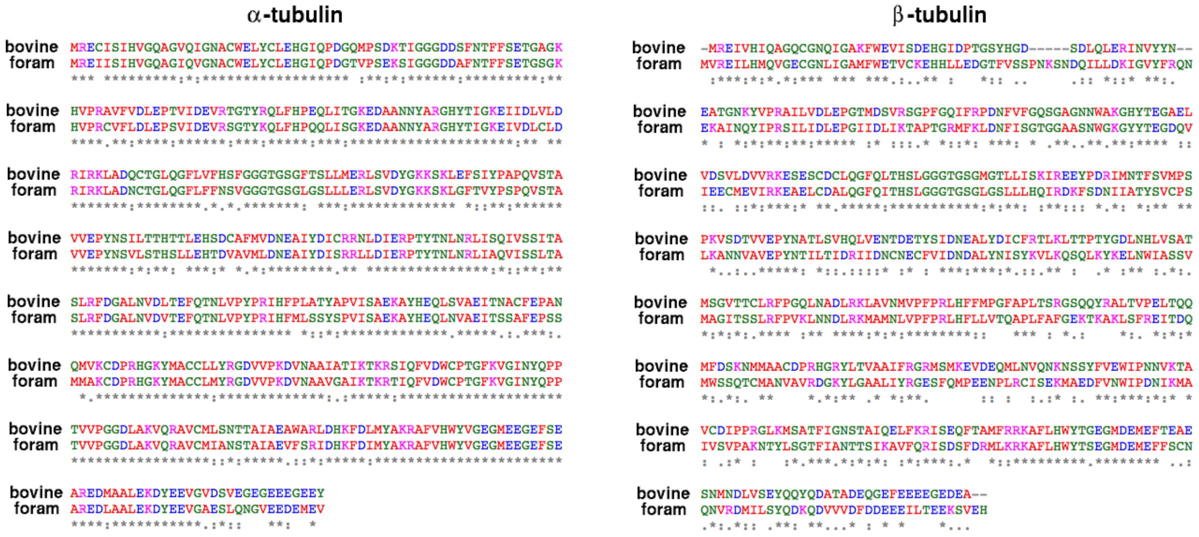

Figure 5: **Sequence alignment of bovine and foram tubulin sequences.** A Clustal Omega 1.2.1 sequence alignment of α- and β-tubulin sequences from Bos taurus (labeled bovine) and Reticulomyxa filosa (labeled foram). The sequence identities are 82% for α-tubulin and 45% for β-tubulin.

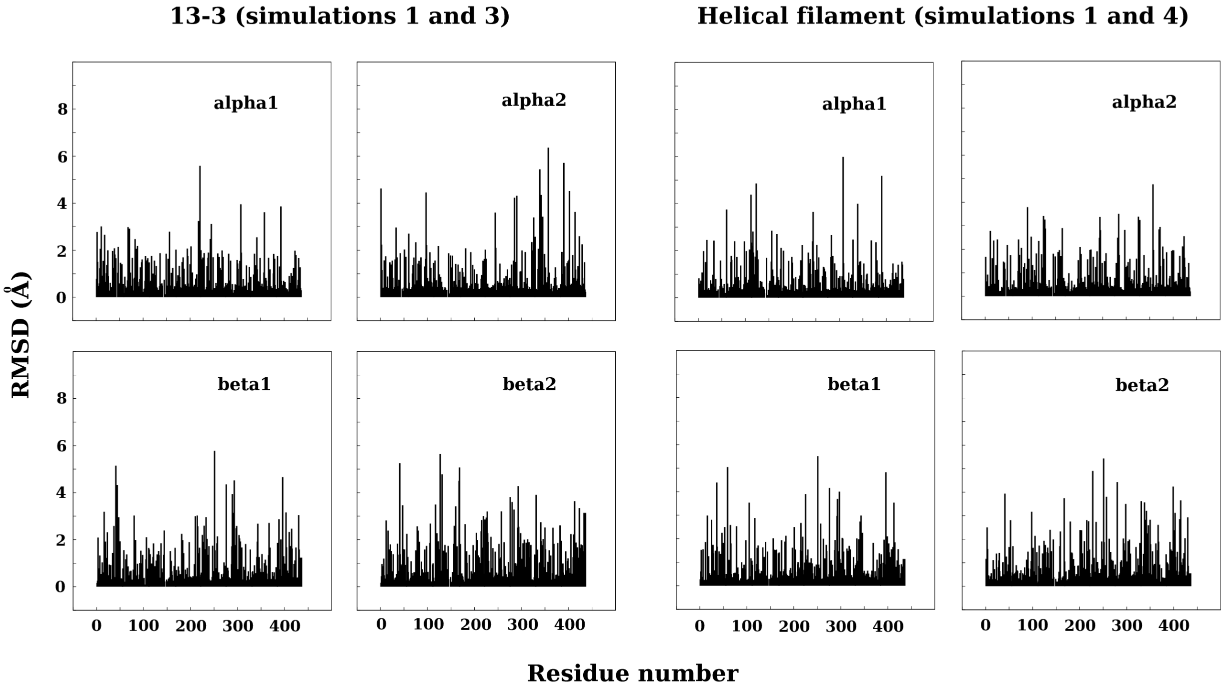

Figure 6: **Comparison of sidechain root mean square deviations (RMSDs) in final structures from two 13-3 and two helical filament simulations that show subtantially different electrostatic interaction energy averages.**

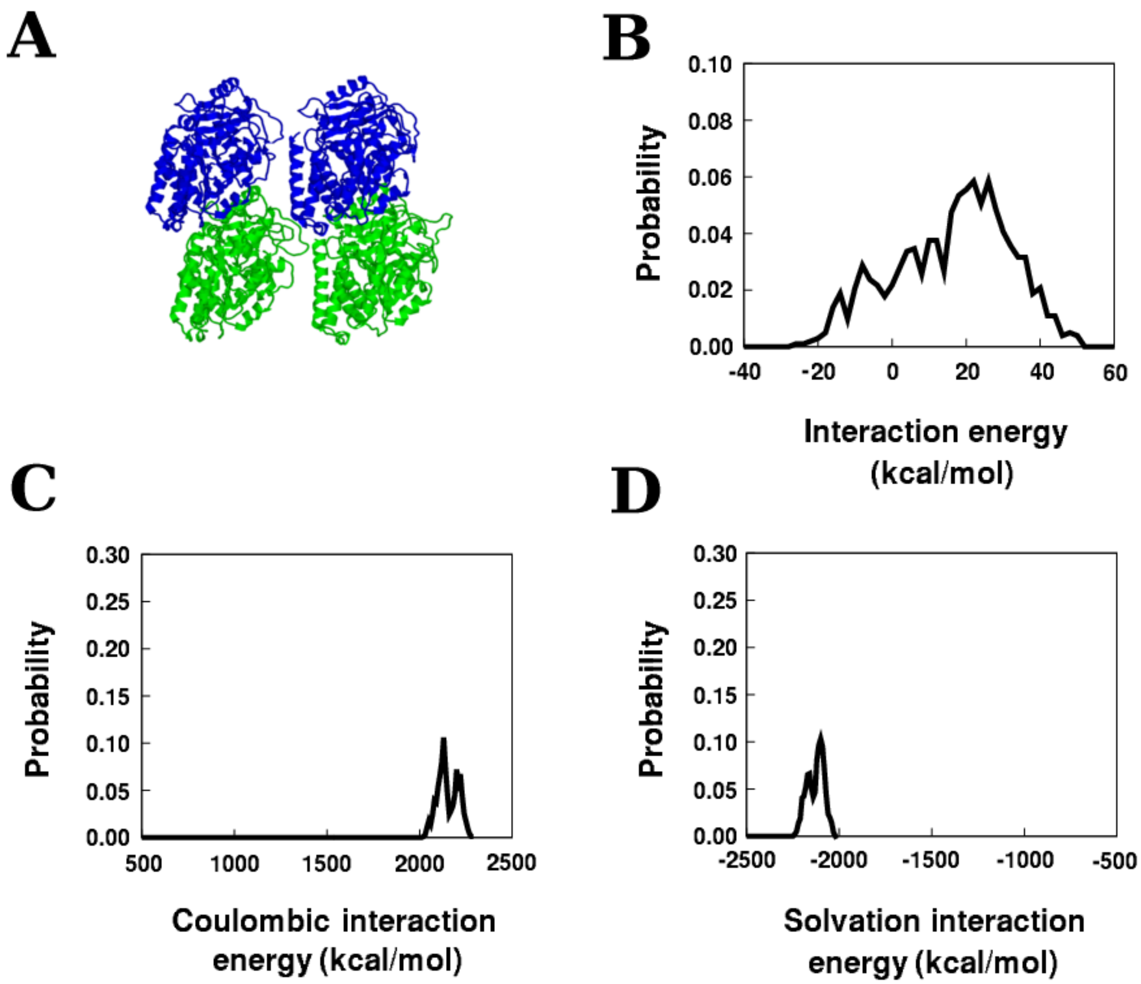

Figure 7: **A decoy foram tubulin inter-dimer interface and its electrostatic interaction energy distributions.** (a) A decoy foram tubulin tetramer with an incorrect inter-dimer interface obtained from the average helical filament model prior to cylindrical restraint optimization. This model complies with the coarse-grained restraints (distances or angles or dihedrals between monomer centers-of-mass), but has an incorrect orientation of the tubulin dimers with respect to the central helical axis, and with respect to each other; (B) Overall electrostatic interaction energy distributions for this interface; (C) Coulombic inter-dimer interaction energy distributions for this interface; (D) Solvation electrostatic inter-dimer interaction energy distributions for this interface. Interaction energies were obtained for a total sampling time of 1 ns for the decoy tetramer (pooled from five separate 0.2 ns simulations), and the probability distributions were generated as histograms with a bin width of either 2 kcal/mol or 10 kcal/mol.
